# Self-Reported Pain Intensity with the Numeric Reporting Scale in Adult Dengue

**DOI:** 10.1371/journal.pone.0096514

**Published:** 2014-05-01

**Authors:** Joshua G. X. Wong, Victor C. Gan, Ee-Ling Ng, Yee-Sin Leo, Siew-Pang Chan, Robin Choo, David C. Lye

**Affiliations:** 1 Institute of Infectious Disease and Epidemiology, Tan Tock Seng Hospital, Singapore, Singapore; 2 Saw Swee Hock School of Public Health, National University of Singapore, Singapore, Singapore; 3 Lee Kong Chian School of Medicine, Nanyang Technologicial University, Singapore, Singapore; 4 Yong Loo Lin School of Medicine, National University of Singapore, Singapore, Singapore; 5 Stata Users Group, Singapore, Singapore; 6 Department of Mathematics and Science, La Trobe University, Melbourne, Victoria, Australia; 7 Singapore Institute for Clinical Sciences, Brenner Centre for Molecular Medicine, Singapore, Singapore; University of Malaya, Malaysia

## Abstract

**Background:**

Pain is a prominent feature of acute dengue as well as a clinical criterion in World Health Organization guidelines in diagnosing dengue. We conducted a prospective cohort study to compare levels of pain during acute dengue between different ethnicities and dengue severity.

**Methods:**

Demographic, clinical and laboratory data were collected. Data on self-reported pain was collected using the 11-point Numerical Rating Scale. Generalized structural equation models were built to predict progression to severe disease.

**Results:**

A total of 499 laboratory confirmed dengue patients were recruited in the Prospective Adult Dengue Study at Tan Tock Seng Hospital, Singapore. We found no statistically significant differences between pain score with age, gender, ethnicity or the presence of co-morbidity. Pain score was not predictive of dengue severity but highly correlated to patients’ day of illness. Prevalence of abdominal pain in our cohort was 19%. There was no difference in abdominal pain score between grades of dengue severity.

**Conclusion:**

Dengue is a painful disease. Patients suffer more pain at the earlier phase of illness. However, pain score cannot be used to predict a patient’s progression to severe disease.

## Introduction

The World Health Organization (WHO) reported that over 40% of the global population is at risk of dengue and an estimated 390 million dengue infections happen worldwide every year.

Dengue incidence has increased and its geographic range expanded.[1] Many studies on the clinical features of dengue were pediatric cohorts.[2], [3] In Singapore, dengue infections have shifted from a primarily childhood to an adult disease. [4] Today, more than 90% of dengue patients are adults. We previously identified the occurrence of bleeding, lymphopenia, hypoproteinemia and elevated serum urea as independent predictors for dengue hemorrhagic fever (DHF) [5].

Pain is an important component of dengue diagnostic criteria. In WHO 1997 guideline, probable dengue requires the presence of fever with any two of headache, retro-orbital pain, mylagia, arthralgia, rash, positive tourniquet test or or hemarroghic manifestation. [6] In WHO 2009 guideline, the diagnostic criteria were revised to comprise fever and two of nausea/vomiting, rash, aches and pains, positive tourniquet test, leucopenia and any warning sign. Warning signs include abdominal pain or tenderness, persistent vomiting, clinical fluid accumulation, mucosal bleeding, lethargy, hepatomegaly and hematocrit rise with rapid platelet count drop [7].

Higher prevalence of abdominal pain, backache, headache and myalgia was noted in adult dengue patients.[3], [8], [9] Abdominal pain and myalgia were associated with dengue severity and mortality.[10]–[12] While these studies identified aches and pains as an important factor for dengue diagnosis and prognosis, we have not found any study assessing the degree of pain reported by dengue patients in predicting progression to severe disease to date. To fill the lacuna in literature, we conducted a prospective cohort study to determine the relationship between pain intensity and disease severity.

## Methods

### Ethics Statement

Ethical approval was provided by the Domain Specific Review Board of the National Healthcare Group, Singapore (DSRB/E/2009/432). Written informed consent was obtained from all subjects.

### Patient Cohort

Acutely febrile patients above the age of 18 were recruited prospectively from January 2010 to September 2012 at the Communicable Disease Center, Tan Tock Seng Hospital, Singapore, a tertiary referral infectious disease centre. All dengue diagnostic testing was performed by the Enviromental Health Institute, a WHO Collaborating Center for Reference and Research on Arbovirus and its Associated Vectors in Singapore. Patients were classified as having laboratory-confirmed dengue according to WHO standards if they were RT-PCR or NS1 positive using the dengue NS1 antigen (Ag) strip (Bio-Rad Laboratories, Marnes-la-Coquette, France).[13] Only laboratory-confirmed dengue patients were included. Detailed daily clinical and laboratory data, including pain score, were collected prospectively until discharge or at the recovery phase of illness. Pain score for all patients, including those who did not return for subsequent follow-ups, were included to increase the sample size.

The Numeric Rating Scale (NRS) is a widely used tool to assess patients’ pain level in chronic and acute illnesses with good responsiveness and sensitivity.[14]–[16]. The NRS was the preferred pain rating scale owing to its ease of use and accurate assessment.[17] The scale ranges from 0 to 10 with 0 being no pain and 10 being the worst possible pain; this is aided with the use of the face pain scale of facial expressions at pain scale of 0,2,4,8 and 10. [18] Separate information on abdominal pain score was captured as a warning sign in the WHO 2009 guideline. On enrolment, patients were asked to rate their pain intensity from day of illness to day of enrolment. Pain scores were then recorded prospectively on subsequent follow-ups.

### Outcome Variable

The primary outcome was the severity of dengue: 1: Dengue fever, 2: Dengue hemorrhagic fever (DHF; Grades I–II) [6] and 3: Severe disease, defined as fulfilling dengue shock syndrome (DSS) and severe dengue (SD) [7].

### Statistical Analysis

Ordinal logistic regression was used to determine how pain score was associated with age, gender, co-morbidity and ethnicity (1: Chinese 2: Malay 3: Indian 4: Others). Generalized structural equation model (GSEM) was applied as a more confirmatory analysis to analyze the data accounting for temporal data structure and the complex dependence among pain score, fever and the outcome.[19] As the latest development in structural equation modeling which unifies regression models with latent class models, GSEM is deemed to be the most appropriate in the proposed data analysis. It could estimate the direct and indirect effects of pain score and fever on the outcome with a single model set-up. In conventional regression analysis one may have to construct multiple models for dealing with studies involving a final outcome, a set of covariates and some variables that are both predictors for the final outcome and intermediate outcomes.

**Figure 1:**
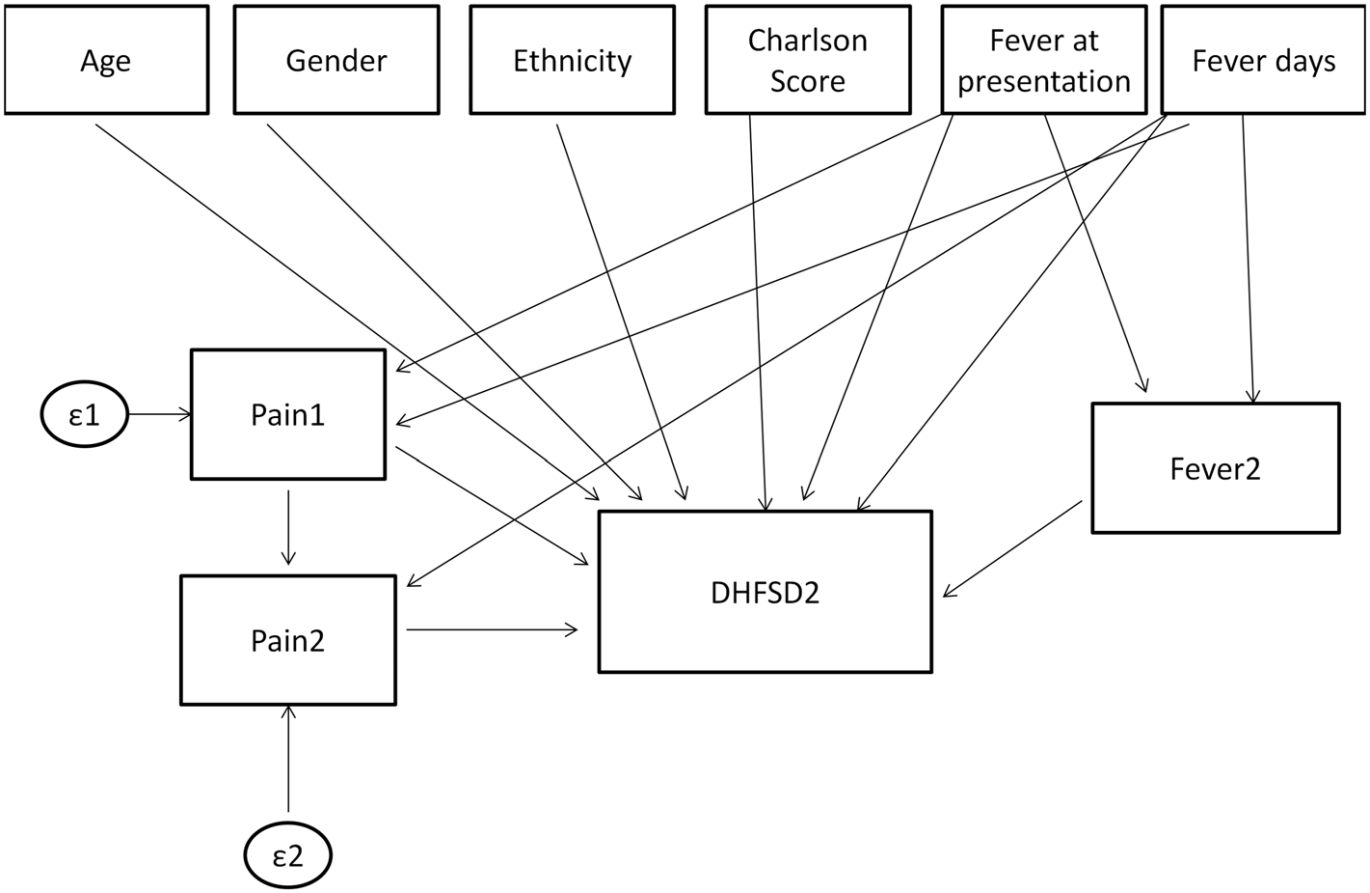
Model specification for study day 2. Feveratpresentation = Fever present at enrolment (Yes/No), Feverdays = Number of fever days up to enrolment, Fever2 = Fever on study day 2, Pain1 = Pain Score at enrolment/presentation, Pain2 = Pain Score on study day 2, DHFSD2 = Outcome on study day 2.

**Figure 2:**
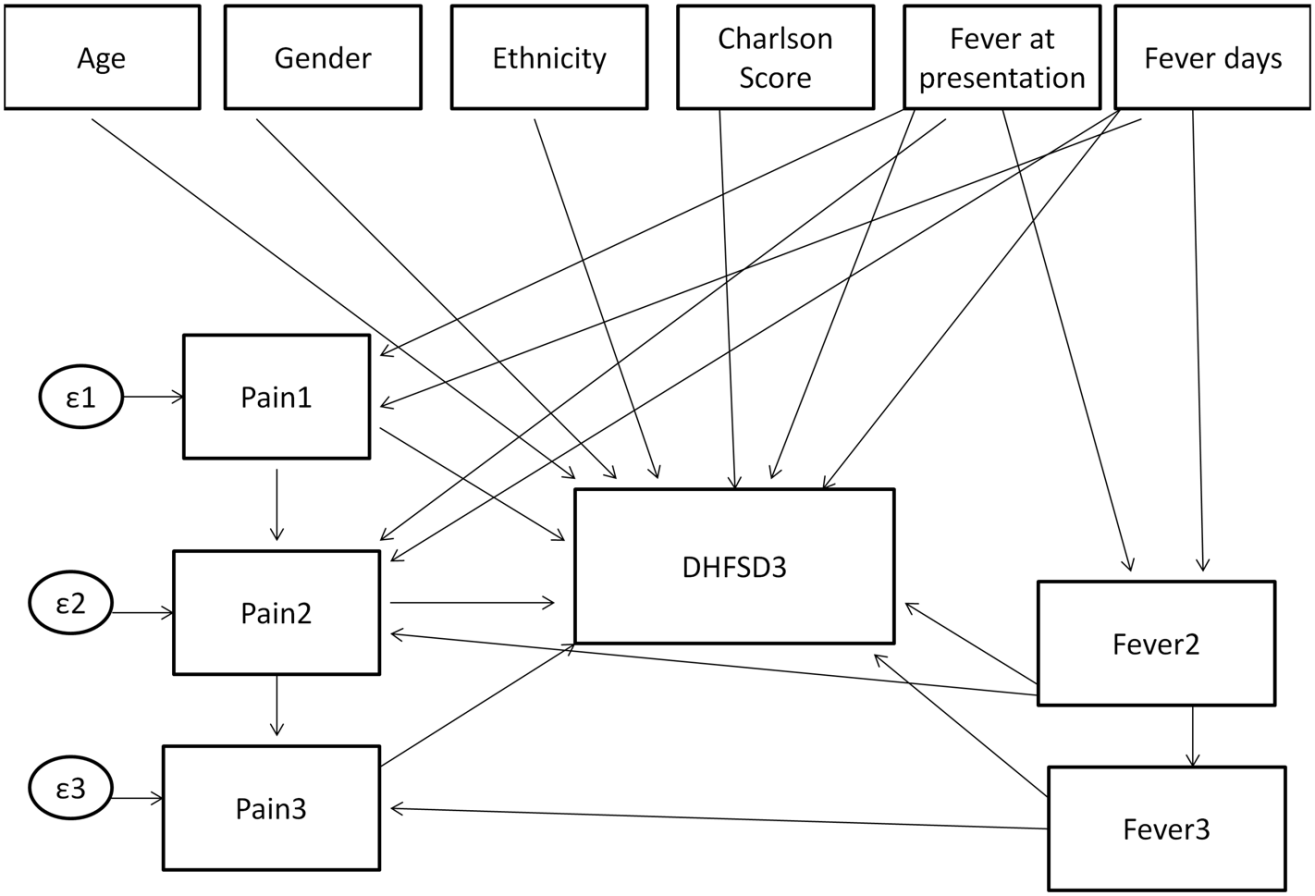
Model specification for study day 3.

Two sets of analyses were carried out, with the outcome ascertained on day 2 and day 3 of enrolment. In both analyses, the number of fever days reported by patients preceded fever at presentation which in turn preceded days 2 and 3 of enrolment (see Figures 1 and 2).

The first GSEM model to predict dengue severity on the second day of enrolment (day 2) involved baseline covariates such as age, gender, ethnicity, Charlson’s co-morbidity score[20], fever at presentation and number of fever days before day 2 as the direct effects to the outcome. The intermediate variables involved whether patient had fever on day 2, pain score on study days 1 and 2. Fever at presentation and the number of fever days were hypothesized to be associated with pain score on day 1 and fever on day 2. Pain score on day 1 was assumed to be associated with pain score on day 2 (Figure 1). To predict dengue severity on the third day of enrolment (day 3), the model was extended to accommodate the pain score and fever on day 3 (Figure 2). The GSEM is most appropriate when such data structures with irreversible temporal effects are required in analysis.

Estimated with maximum likelihood, the models were analysed with the multinomial distribution and logit link. Analyzed with Stata MP 13 (Stata Corp, Texas, USA), all statistical tests were conducted with 95% confidence intervals.

## Results

### Demographic Description of Cohort

Of the 850 patients recruited, 499 tested positive for RT-PCR or NS1 were included in the analysis. There were 111 DHF I–II and 13 DHF III–IV(DSS) cases according to WHO 1997 classification. Twenty were severe dengue cases in accordance with the WHO 2009 classification. The median duration of fever was 6 (3–9) days. One patient died owing to myocarditis. One patient was diagnosed with both DSS and SD. In total, 32 patients fulfilled the severe disease classification.

The median age of the cohort was 34 years (5^th^–95^th^ percentile, 21–51 years). Male patients comprised 79% and 77.6% of the cohort were Chinese. Medical co-morbidity, mainly myocardial infarction, diabetes and cerebrovascular dieseases was present in 1.8%. The majority (76.3%) of patients had fever on the first visit. The median pain score was similar in DF, DHF I–II and severe disease patients (5 [0–8], p = 0.7). (Table 1) There were no significant statistical association between pain score at first visit and age (p = 0.09), gender (p = 0.32), co-morbidity (p = 0.49) or race (p = 0.19).

**Table 1:** Demographic description of study cohort.

	DF (n = 356)	DHF I–II (n = 111)	Severe disease (n = 32)	Overall (n = 499)
Age in years	32 (21–46)	40 (22–58)	36 (23–57)	34 (21–51)
Male gender	289 (81.1%)	89 (80.2%)	18 (56.2%)	396 (79.4%)
Chinese ethnicity	258 (72.5%)	103 (92.8%)	26 (81.2%)	387 (77.6%)
Any co-morbidity	5 (1.4%)	3 (2.7%)	1 (3.1%)	9 (1.8%)
Fever at 1^st^ visit	250 (70.2%)	100 (90.1%)	31 (96.9%)	381 (76.3%)
Fever days at 1^st^ visit	6 (3–9)	6 (3–8)	5 (3–9)	6 (3–8)
Pain score at 1^st^ visit	5 (0–8)	5 (0–8)	5 (0–8)	5 (0–8)

For categorical variables, absolute numbers (and the relative percentage) are being indicated. For continuous variables, medians (and the relative 5^th^–95^th^ percentile) are being indicated.

### Temporal Trend of Overall Pain Score

**Figure 3:**
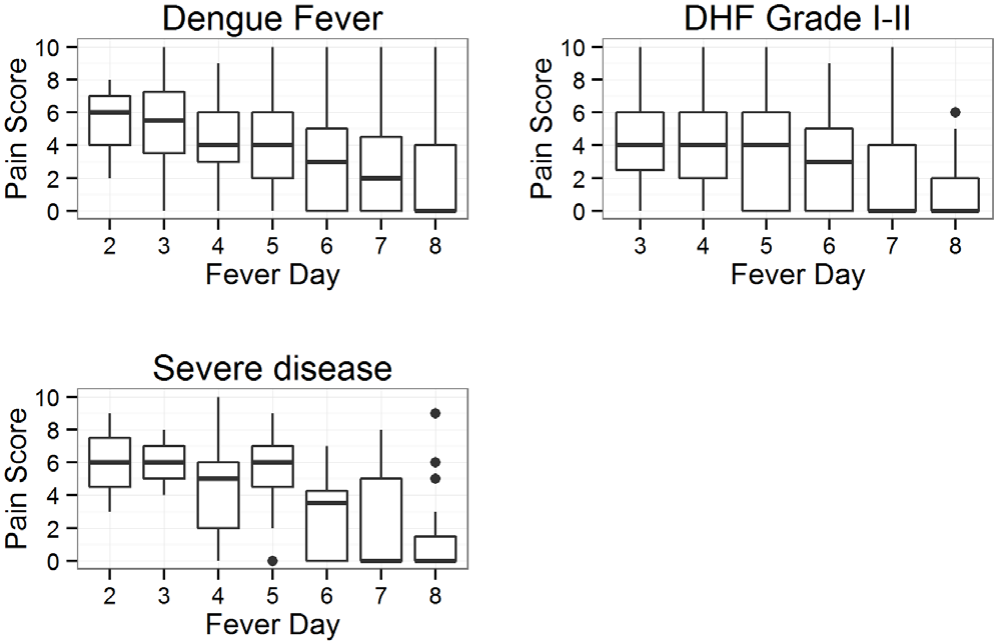
Boxplot of pain scores over fever days by dengue severity. The line inside the box represent the median, box edges represent 25^th^ and 75^th^ percentile and vertical lines represent 1.5 times the interquartile range. Points outside these limits are considered outliers.

Figure 3 shows that DF patients generally experienced reduction in pain as they were recovering from dengue infection. Median pain score for patients with DHF I–II or severe disease overlapped. However, pain score diminished to the range of 0–2 on fever day 8. It is visually difficult to draw conclusions on differences between dengue severity from the plots due to the large overlap. (*For detailed sample sizes for each day, refer to Table S1).*

### Predictive Modeling of Overall Pain Score and Co-variates

#### Study day 2

Pain score on study days 1 and 2, number of fever days, ethnicity and co-morbidity were not significantly associated with dengue severity (Table 2). The GSEM results showed that age and fever at presentation were predictive of severe disease on study day 2. On average patient’s risk of severe disease increased by 3% with every year of increase in age. In addition, fever at presentation increased the risk of developing severe disease (Table 2). Patients experienced less pain as they recovered (odds ratio [OR]: 1.52 [1.33–74]).

**Table 2:** GSEM results for likelihood of developing severe dengue disease on days 2 and 3 of enrolment.

Baseline co-variates	Study Day 2 (n = 438) aOR (95% CI)	Study Day 3 (n = 399) aOR (95% CI)
Age	1.03 (1–1.06)*	1.05 (1.00–1.10)*
Gender		
Male	Reference	
Female	1.48 (0.68–3.23)	1.08 (0.32–3.65)
Ethnicity		
Chinese	Reference	
Malay	0.59 (0.07–4.96)	2.14 (0.15–30.07)
Indian	0.14 (0.02–1.06)	0.24 (0.03–1.95)
Others	0.59 (0.07–4.90)	2.70 (0.24–30.73)
Charlson’s co-morbidity score		
0	Reference	
≥1	1.51 (0.25–8.91)	1.56 (0.12–21.20)
Fever at presentation		
No	Reference	
Yes	6.76 (1.41–32.4)*	0.77(0.17–3.60)
Fever duration in days	1.29 (0.96–1.73)	1.27 (0.82–1.97)
Fever on Study day 2		
No	Reference	
Yes	1.93 (0.85–4.37)	0.54 (0.18–1.62)
Fever on Study day 3		
No	NA	Reference
Yes	NA	3.97 (1.18–13.34)*
Pain score at presentation	0.96 (0.83–1.10)	1.09 (0.90–1.32)
Study Day 2 pain score	0.96 (0.82–1.13)	1.15 (0.92–1.42)
Study Day 3 pain score	NA	3.97 (1.18–13.34)*

*Statistically significant; equivalent to p<0.05.

#### Study day 3

Similarly, pain score on study day 2 was not predictive but age and fever on day 3 of study were predictive of dengue severity. The risk of age on severe disease largely remained the same as the day before. In addition, fever on day 3 increased a patient’s risk of severe disease. Notably, patients who developed severe disease on day 3 were more likely to report higher pain scores (Table 2).

### Abdominal Pain

Ninety five patients (19%) experienced abdominal pain. Four of them had SD while the other 3 had DSS. Of these 7 cases, 3 of them experienced the worst abdominal pain on the same day as they were diagnosed with severe disease or after progressing on to severe disease. The median abdominal pain score for patients with severe disease was 5 (5^th^–95^th^ percentile, 2–9) and 4 (5^th^–95^th^ percentile, 1–10) for non-severe disease (p = 0.38).

## Discussion

Abdominal pain was identified as one of four warning signs in a retrospective Puerto Rican study on dengue deaths.[21] In Cuba, headache was present in all twelve fatalities.[22] In Singapore abdominal pain was present in half of adult dengue deaths.[23] In Lucknow, India, severe abdominal pain was noted in 18% of children and adults with dengue shock syndrome[24]. In the Philippines, abdominal pain was significantly more common in children with DHF.[25] Abdominal pain was significantly associated with the need for major interventions in the DENCO study [26] while in Brazilian children, abdominal pain was independently associated with severe dengue.[27] Notably, be it dengue shock syndrome or uncomplicated dengue, headache and abdominal pain were significantly more common in adults compared with children [3].

To our knowledge, this is the first study to analyze the utility of a numeric pain score in predicting the progression of dengue severity. We explored the utility of pain score in dengue prognosis accounting for baseline covariates which might affect pain. The results from the GSEM model should not be interpreted as a predictive model for dengue disease severity. We found that pain intensity measured by numeric rating scale experienced by adult dengue patients did not differ by ethnicity, co-morbidity and dengue severity. Additionally abdominal pain intensity did not differ by dengue severity. Age and fever at presentation appeared to correlate with dengue severity. The impact on age on dengue severity was previously studied [28].

There were several limitations in our study. We were unable to monitor patients’ parameters before enrolment. Our patient profile at a tertiary center may not reflect those in primary care. Patients were recruited at a median of 6 days of fever. The daily level of pain by recall before enrolment can be unreliable and may be associated with recall bias. Patients who visited the clinic only on enrolment day were included in the analysis for increasing the sample size; we assumed that patients were most painful on enrolment day and subsequent follow-up were missed as they recovered. Our study was conducted in an adult cohort which may not generalize to children with dengue. Further work needs to be done to monitor level of pain in the early course of illness to better understand the relationship between pain and disease progression in dengue.

## Conclusion

This study provides evidence for clinicians against using pain score alone in clinical triage or in predicting progression to severe disease.

## Supporting Information

Table S1 Sample size at each fever day stratified by outcome (DHF I–II = Dengue hemarroghic fever grades 1–2).
(DOC)
