# Supplementary material for: Self-Reported Pain Intensity with the Numeric Reporting Scale in Adult Dengue
Source: PLoS One. 2014 May 1;9(5):e96514. doi: 10.1371/journal.pone.0096514 (PMC4006847; doi:10.1371/journal.pone.0096514)
Supplement: Table S1 — Sample size at each fever day stratified by outcome (DHF I–II = Dengue hemarroghic fever grades 1–2). (DOC) [file pone.0096514.s001.doc]

Table S1: Sample size at each fever day stratified by outcome (DHF I-II = Dengue hemarroghic fever grades 1-2).

| Fever Day | Dengue fever | DHF I-II | Severe disease |
| --- | --- | --- | --- |
| 2 | 4 | 0 | 2 |
| 3 | 22 | 13 | 3 |
| 4 | 72 | 32 | 9 |
| 5 | 136 | 62 | 17 |
| 6 | 191 | 96 | 22 |
| 7 | 246 | 119 | 28 |
| 8 | 220 | 123 | 29 |
